# Exploring multiple levels of suffering and suicide prevention in an era of emerging national legislations

**DOI:** 10.3389/fpubh.2025.1589426

**Published:** 2025-06-24

**Authors:** Mark Goldblatt, Dolores Angela Castelli Dransart, Sylvie Lapierre, Margda Waern, Reinhard Lindner

**Affiliations:** ^1^Harvard Medical School, Department of Psychiatry, Boston, MA, United States; ^2^Mass General Brigham, McLean Hospital, Belmont, MA, United States; ^3^HES-SO University of Applied Sciences and Arts Western Switzerland, School of Social Work, Fribourg, Switzerland; ^4^Department of Psychology, University of Quebec, Trois-Rivières, QC, Canada; ^5^Center for Research and Intervention on Suicide, Ethical Issues and End-of-Life Practices (CRISE), Montreal, QC, Canada; ^6^Department of Psychiatry, Institute of Neuroscience and Physiology, University of Gothenburg, Gothenburg, Sweden; ^7^Department of Psychotic Disorders, Sahlgrenska University Hospital, Region Västra Götaland, Gothenburg, Sweden; ^8^University of Kassel, Faculty of Humanities, Institute of Social Work, Kassel, Germany

**Keywords:** assisted dying, suicide, assisted suicide, medical aid in dying, suffering

## Abstract

Access to assisted dying (variously called assisted suicide, euthanasia, and medical aid/assistance in dying) is increasingly available in many countries around the world. Individual suffering in the present and fears for the future, feature prominently in the request for assisted dying, which then affects many people beyond the identified individual, including family and loved ones, the clinical team associated with the dying process and societies. We consider expanding waves of distress, beginning with the subjective intrapsychic suffering of the individual requesting assistance in dying, the interpersonal effect on survivors, and the complex responses in those facing requests for assisted death. The autonomous rights of the individual to the alleviation of suffering are balanced by efforts toward suicide prevention, which are impacted when there are limited options to alleviate the psychosocial and existential suffering of those who express the wish to die.

## Introduction

The legalization or depenalization of the process in assisting the death of another person is increasing in countries across the world. Assisted suicide describes the process by which the dying person ends their own life with the assistance of others, e.g., physicians, and differs from euthanasia, in which the act is carried out by a person other than the dying person, at the dying person’s request through legally sanctioned methods. In some countries assisted suicide and euthanasia are subsumed under the term of medical assistance/aid in dying (MAID) or assisted dying. Suicide commonly refers to death by one’s own means, with no assistance from others.

Multiple factors influence individuals, particularly older adults, to consider assisted dying, including unbearable physical suffering/pain, psychological, and social issues as well as cultural circumstances (1). In some countries a trend has emerged to consider assisted suicide and even euthanasia as a human rights issue and separate it conceptually from suicide. Although there are overlapping areas between suicidal people and those requesting assisted death, especially among the older adult population, there are also significant differences, especially relating to how others are involved in the death. People in both circumstances appear to be in a distressed state, worried about pain and incapacity. Those requesting assisted dying are usually approaching the end of their natural life, due to incurable physical problems, and involve others, like family and physicians in a legally recognized decision to hasten death in a manner acceptable to society. Suicide is often driven by psychopathology, interrupts the course of life, and is often undertaken alone. In both cases, death deeply affects relationships (survivors), communities and treaters (2).

In this paper, we consider assisted dying and multiple levels of suffering in four different countries, drawing from clinical and research data in the USA, Sweden, Canada (Quebec), and Switzerland. We first focus on consideration of the internal psychological experience of individuals requesting assisted death; we then consider the experience of older adult’s accessing health care in Sweden; we compare people requesting Medical Aid in Dying (MAID) and suicidal individuals in Quebec, Canada and we end with a description of the experience of professionals dealing with requests for assisted suicide in older adults in Switzerland.

### The internal experience of people requesting assistance in suicide

Requests for assisted dying are complex and are influenced by external and internal factors. Even in the terminally ill or geriatric population these requests are invariably ambivalent (3) and are linked to thoughts and feelings relating to concerns about palliative and end-of-life care, as well as internal conflicts around fears of pain and dependency. Patients facing end-of-life often experience intense affects of anguish, hopelessness, rage, and guilt. In addition, particular kinds of (unconscious) fantasies often feature prominently in suicidal states, with themes of abandonment, revenge, rebirth, and self-punishment (4). These fears may mobilize suicidal plans, as a way of avoiding the conflictual emotions that arise. The terminally ill patient might say, “I cannot live like this. I need to be in control of my body. I want to be able to take my life when I feel the time is right” which reflects of an underlying fear; “No one will be there to help me when the illness becomes more than I can stand. I will be left alone to suffer without help or caring.” The fear of isolation and loss in the time of dying often drives the desire for an assisted death. Good palliative care aims to restore a sense of autonomy and increase quality of life, by controlling debilitating symptoms and thereby reducing psychological distress, including the sense of helplessness and loss of control, as death is faced with realistic fear and support.

The decision to request assistance in dying is often complicated by intense affective states of guilt and shame. Further complications arise when psychiatric illnesses, such as Major Depression are present, even though they may be difficult to diagnose in end-of-life states. External influences, such as the wishes (real or imagined) of important family and friends also play an important role.

To understand the complexity of the request for assisted suicide we should consider conscious and unconscious factors and motives that lie behind apparently rational requests for assisted death.

For example, the request for assistance in dying is not only a plea to reduce suffering, but may also have a relational meaning, as a way to deal with unresolved interpersonal struggles and internal conflicts (3, 5). The assertion of the right-to-die can also mean, an attempt to gain control, and avoid the terrors and desperation of dying (6). The meaning of the communication of a request for assisted death can therefore be understood as a complex reaction to the fear of death: a desperate fear of dependence, loss of control, helplessness and shame, associated with feeling overwhelmed as physical, cognitive and emotional resources are diminished and early, unresolved developmental issues become reactivated.

### A Swedish perspective on older adults’ physical conditions, pain and suicidality and their interactions with health care providers

Assisted dying is currently illegal in Sweden. However, there is a long research tradition focused on the health and well-being of older adults, which contributes to the understanding of the wish to end life in this age group. We identify five studies that describe important aspects of distress of the older adult population.

A study involving pooled population-based data from eleven European countries (7) underscored the impact of physical functioning on the wish to die by showing a “dose–response” between level of functional loss and wish for death in persons aged 65 and above. A literature review on physical illness, functional disability, and suicidal behavior among older adults over 65 (8) found that in addition to functional disability, a number of specific medical conditions were associated with suicidal ideation and behavior. A third study (9) underscored how pain greatly affects the quality of life and the wish to live, possibly due to increased feelings of burdensomeness, reduced fear of death, and the development of hopelessness, depression and unbearable psychological pain.

A qualitative research study with older adults who survived a suicide attempt (10) showed a superordinate theme summarized as *Loss of self,* and two subthemes of feeling devalued; one was *Self-devaluation,* and the other was *Feeling devalued by health care providers*. Implicit ageism on the part of medical care providers appears to have contributed to some of the negative experiences that impacted the older patient’s decision to end life. Coordination of care services was lacking, making it even harder to navigate the health care system. Interventions to alleviate both psychological and physical symptoms, including relevant medication adjustments and referrals for surgical procedures allowed the individual to then shift their focus back to living (10).

A study on the fluctuating nature of suicidal ideation in older adults found that participants who had initially reported having had suicidal feelings during the week prior to an examination, found that almost half (45%) reported no such feelings subsequently (11) which is an important consideration when discussing health care options for older adults who have lost the will to live.

The results from both clinical and population-based studies suggest a need for inclusive dialog between older adult patients and their health care providers. Without such an opportunity, older adults with physical illness, functional limitations and pain, may not be able to make truly informed decisions about living or dying as they consider death either by their own hand, or with the aid of a physician.

### Similarities and differences in suffering between persons who request medical aid in dying and those who think about suicide. The situation in Quebec, Canada

Quebec, Canada has a very high proportion of death by Medical Aid in Dying (MAID) accounting for 7% of all deaths in the province (12). Although unbearable and irremediable suffering is the cornerstone of all MAID legislation, and extends beyond physical pain, legislators have failed to adequately define this concept, leaving these key terms open to interpretation (13). This absence of clarity is particularly problematic given that the notion of suffering is multidimensional, contested, and inconsistently described in scholarly literature (13). To understand this situation, researchers and clinicians tried to compare the suffering and motives for death between people who think about suicide and those who request MAID.

The comparison between these two groups shows that both experienced multiple losses. Motives for dying were similar in both groups, expressed as unbearable physical and psychological suffering, associated with a combination of social, psychological, and existential issues that were intensified by their medical context. The inability to adjust to major changes resulted in a sense of being trapped in a perceived hopeless and humiliating situation, leading to feelings of helplessness, despair, apprehension about the future, and a lack of meaning to their lives (13–15). Both groups perceived themselves as a burden for others (16–18). They viewed death as the only solution to end their suffering because they believed their situation was beyond improvement.

There were some notable differences between individuals requesting MAID and those who are thinking about ending their life (19). Usually, people requesting MAID are approaching the end of natural life due to incurable physical problems; while suicide which is often driven by psychopathology, interrupts the course of life. However, with the expansion of MAID to individuals who are not at the end of life, the distinction based on life stage and type of suffering becomes less clear. Unlike suicide, MAID is a highly collaborative process where patients involve their physician and, most often their family (though the final choice ultimately rests on the individual), in a legally recognized, and socially acceptable, decision to hasten death (19). However, offering MAID to a patient who has not raised it could be interpreted as an indication that their suffering will likely become intolerable, and that MAID is the recommended way out, impacting a patient’s hope and resilience (20).

### Challenges of Swiss professionals facing the request for assistance in dying

A request for assisted dying raises questions and concerns for the professionals involved in providing end-of-life care, in particular, whether assisted dying should be considered a health care intervention (21) and whether assisted dying is compatible with their professional ethos, identity and personal values (22). Professionals’ attitudes in regard to assisted dying vary. In most studies examined in the meta-review by Quah et al. (23), professionals did not endorse assisted dying which created moral or professional dilemmas. They felt they were not competent to be involved in such practice (2) or feared the emotional impact of this act (2, 24, 25).

In a Swiss study (22) of professionals working in institutions for older adults, one fifth of participants reported being significantly affected, on multiple levels, when faced with a request for assisted dying. On a personal level they reported experiencing strong emotions, general distress, and anxiety. On a professional level they questioned their professional ethos and struggled to reconcile professional values such as providing appropriate care with respecting the individual’s end-of-life choices. On an organizational level, they reported effects on the operation of the institution. Approximately half of the respondents felt that assisted suicide was compatible with their professional purpose and practice, but did not want to be the one to provide assistance in dying. For 16 out or the 40, assisted dying was not compatible with their professional purpose, but they agreed to accompany the patient for the sake of continuity of care or because they valued the relationship with the individual making the request. A minority, (3/40), refused to be involved, activating the conscientious objection clause, as they considered assisted dying to be totally contrary to their personal and professional ethos.

Once the person’s decision was finalized, care professionals experienced that the relationship with the older adult changed. In some situations, it could deepen and come to closure. At other times, the relationship was disrupted to the point that communication and physical proximity for care became difficult. They felt that in order to perform in a manner that is both professional and humane, they needed consistent support and opportunities in real time for emotional venting, ethical deliberation and personal and collective elaboration of the process in which they are involved.

## Discussion

People often experience great psychological and physical suffering as they approach the end of life. Even when good hospice care and palliative treatments are available, the fear of helpless suffering appears to weigh heavily on requests for assistance in dying. In recent years there has been a shift in the sociodemographic and clinical profiles of those who choose assisted dying, weighted towards greater proportions of very old adults, and persons who are depressed and/or isolated.

We propose a model of three levels of suffering in the context of assisted suicide, based on studies of psychological and physical suffering. On the individual level, the intrapsychic experience of unbearable feelings and perceptions result from internal conflicts, relationships, and fears of loss and abandonment. The request for assisted suicide can then be perceived as a solution to these unbearable feelings in the context of the desire for connection and companionship, weighed against the wish for separation and detachment. The second level of suffering concerns the suicidal person’s interpersonal relationship with those close to them, including the professionals involved in their care. A complex interactional process takes place amid this suffering, where those involved influence each other in their attitude and reactions towards assisted suicide. The third level of this analysis focuses on societal attitudes towards aging, dying, and the psychological and physical suffering that accompanies these processes. Suicide may emerge as a perceived solution to existential human conditions from an ageist perspective. This dynamic can also influence the attitude of older individuals towards their own suffering, potentially leading them to seek a radical end to life rather than seeking help for their suffering.

Suffering is an inescapable aspect of life and for both suicidal people and people requesting assisted dying, death is primarily viewed as an end to suffering. However, there are various ways to address suffering. First, it would be important to focus on implementing concrete aids in living (26). Meaning-centered psychotherapy can address psychosocial distress and existential suffering by enhancing a sense of purpose in life, peace and well-being, even as individuals approach the end of life (27, 28). Further training is essential for health care professionals beyond the medical and legal aspects, to include emotional, relational, and symbolic dimensions of assisted death (29). This training should include a segment on ageism, since 85% of those who received MAID in Quebec, were aged 65 and over. Advanced age and the concept of “completed life” appear to justify the use of assisted dying among professionals and older adults alike, who internalize society’s negative perception of aging (30, 31). Simulation training could contribute to effectively preparing healthcare professionals to deal with euthanasia requests and suicidal individuals (32). For suicide prevention workers, a training on assisted dying is also valuable, as many feel uneasy and unprepared to discuss the topic, despite the fact that the majority (76%) have faced situations where the caller who wanted to die was suffering from an incurable illness (33–35). In addition, training on suicide prevention should be offered to all health care professionals assessing requests for assisted death (35, 36), since there appears to be an overlap between the determinants of suicide and requests for MAID (37).

Freedom to choose assistance in dying in those countries where it is legal may underestimate the role of personal and interpersonal suffering that contributes to the request, as though participants were making a rational decision without emotional overlay. We suggest that there is complexity to this request involving interlinking circles of influence, where the inner conflicts of the person requesting assistance in dying interacts with other people involved in the process. These considerations are key to the development of effective suicide prevention strategies and the promotion of societal dialogue on the broader issues of aging, suffering, dying, and the provision of necessary assistance for distressed life circumstances.

### Strengths and weaknesses of this study

In considering multiple layers of suffering in the request for assistance in dying, we suggest a model that offers a fresh framework for understanding assisted dying beyond the traditional biomedical and bio-ethical perspective.

All of the authors share a common research interest in the field of suicidology, which influenced the development of this proposed model. Additional studies are needed to develop a more comprehensive understanding of the wish for assisted death and the effects of suffering on multiple levels, in order to allow this model to be further tested and fleshed out.

## Conclusion

The request for assistance in dying is complex and involves multiple levels of suffering. The individual level of suffering reflects fears of helplessness and shame associated with the anticipated loss of functionality and pain. Interpersonal levels of suffering are reflected in the suffering of family and loved ones, as well as are treaters in the community. The societal implications of assisted dying are reflected in each countries legal system as they try to accommodate wishes for autonomy as well as suicide prevention. The complexity of facing the end of life is supported by relationships and sustenance, worsened by withdrawal and isolation.

## Data Availability

The original contributions presented in the study are included in the article/supplementary material, further inquiries can be directed to the corresponding author.
